# Advances in understanding multifunctionality of *Barley stripe mosaic virus* γb protein

**DOI:** 10.1371/journal.ppat.1013299

**Published:** 2025-07-02

**Authors:** Zhihao Jiang, Meng Yang, Dawei Li

**Affiliations:** 1 State Key Laboratory of Plant Environmental Resilience, College of Biological Sciences, China Agricultural University, Beijing, China; 2 Department of Plant Biochemistry, Center for Plant Molecular Biology (ZMBP), Eberhard Karls University, Tübingen, Germany; Virginia Polytechnic Institute and State University, UNITED STATES OF AMERICA

## Abstract

Plant viruses usually encode versatile but limited number of proteins to reshape the cellular microenvironment, suppress or co-opt host pathways and proteins for their own benefit. *Barley stripe mosaic virus* (BSMV, *Hordeivirus hordei*) is a positive single-stranded RNA virus that infects both monocots and dicots. Among its seven encoded proteins, the γb protein exhibits remarkable multifunctionality despite being the smallest one. By interacting with various viral and host proteins, γb acts as a master regulator participating in almost all steps of the viral life cycle, including replication, movement, virion morphogenesis and vertical transmission, in addition to counteracting several layers of host defenses. In this review, we systematically summarize current understanding of the γb protein multifunctionality and discuss its implications in the ongoing co-evolutionary battle between host plants and invading viruses.

## Introduction

**Fig 1:**
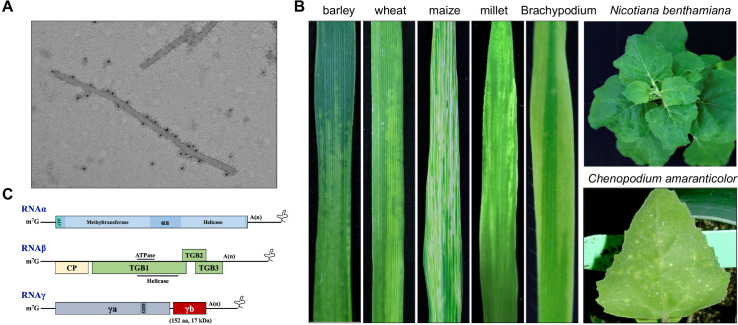
Symptoms and genome organization of *Barley stripe mosaic virus* (BSMV). **(A)** The uniform distribution of γb on BSMV particles, as visualized using the immunogold labeling approach. This panel is adapted with permission from [32]. **(B)** Representative symptoms induced by BSMV on different monocot and dicot plants. **(C)** The BSMV genome consists of three RNAs, designated RNAα, RNAβ, and RNAγ. Each RNA features a 5′ cap structure (m7G) and a 3′ internal polyadenylate sequence.

The genus *Hordeivirus* has been assigned to the family *Virgaviridae* [1], including five species-*Barley stripe mosaic virus* (BSMV, *Hordeivirus hordei* in binomial nomenclature), *Lychnis ringspot virus* (LRSV, *H. lychnis*), *Poa semilatent virus* (PSLV, *H. poae*), *Anthoxanthum latent blanching virus* (ALBV, *H. anthoxanthi*), and *Ligustrum mosaic virus* (LigMV) [2]. As the type member of *Hordeivirus*, BSMV is a single-stranded (ss) positive RNA virus with a rod-shaped virus particle (Fig 1A); it mainly infects barley (*Hordeum vulgare*), occasionally wheat (*Triticum aestivum*) and oat (*Avena sativa*) in nature; it can also infect maize (*Zea mays*), millet (*Setaria italic*), *Brachypoium distachyon*, *Chenopodium amaranticolor*, and *Nicotiana benthamiana* under experimental conditions (Fig 1B). BSMV contains three genomic RNAs, designated RNAα, RNAβ, and RNAγ (Fig 1C). Each of the three genomic RNAs is individually packaged into a distinct rod-shaped particle, which can be differentiated based on its length. RNAα encodes the helicase subunit of the viral RNA-dependent RNA polymerase (RdRp) complex, named αa protein; RNAβ encodes the coat protein (CP) and triple gene block (TGB1, TGB2, and TGB3) responsible for virion assembly and movement; RNAγ encodes the polymerase subunit of the viral RdRp complex, named γa protein and the multifunctional γb protein (Fig 1C). Owing to the orchestrated functional partitioning of replication and movement processes among the three genomes [3,4], BSMV has served as an excellent probe to study the viral infection cycles for several decades.

**Fig 2:**
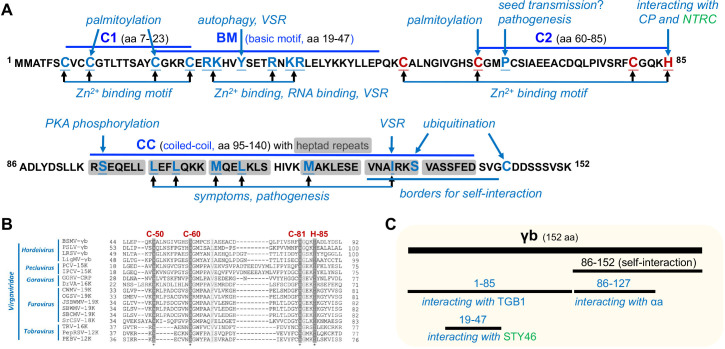
Characterization of the γb protein. **(A)** The key amino acids and motifs contributing to the multifunctionality of the γb protein. **(B)** Amino acid sequence alignment of CRP proteins in different genera of *Virgaviridae.* The conserved CCCH motif (Cys-50, Cys-60, Cys-81 and His-85 for BSMV strain ND18) are highlighted in dark grey. **(C)** A schematic diagram illustrating the interactions between different regions of the γb protein and various proteins.

**Fig 3:**
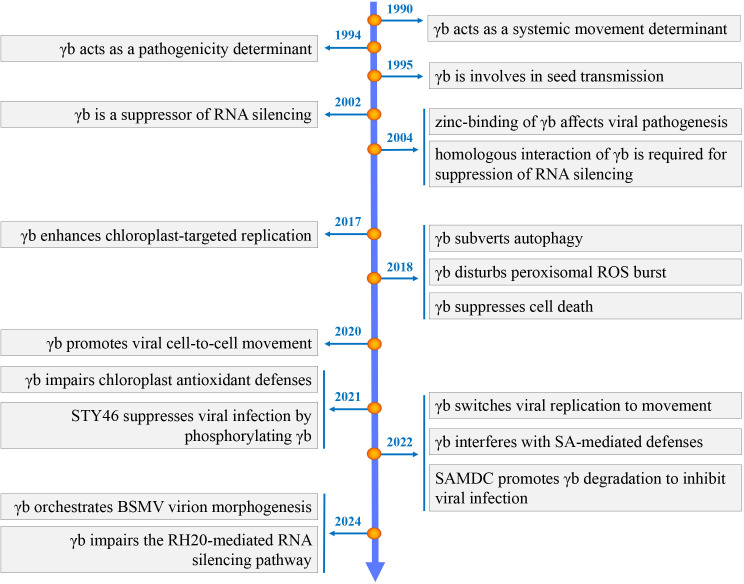
A timeline summarizing progress in understanding the multifunctionality of the BSMV γb protein.

The hordeiviral γb proteins are translated from subgenomic RNAγ, ranging in size from 15.77 to 20.13 kDa and sharing an identity of about 58%. The PSLV γb has the largest molecular weight (approximately 20 kDa), the BSMV and LRSV γb are around 16–17 kDa. Despite the various of length, hordeiviral γb proteins contain four conserved domains, designated C1, basic motif (BM), C2, and coiled-coil (CC) motif (Fig 2A). As the cysteine-rich proteins (CRPs), nine of the eleven cysteines within γb are concentrated in two zinc-finger-like motifs: C1 and C2. Sequence alignment of different CRPs among *Virgaviridae* show a conserved CCCH motif (Fig 2B). The BM motif, which is rich in arginine and lysine, exhibits RNA-binding activity and is involved in the replication process. All three motifs (C1, BM, and C2) have independent zinc-binding activity [5]. Deletions or mutational analyses within C1, BM, and C2 sequences highlight the pivotal role of BSMV γb in pathogenesis, disease development as well as viral accumulation [5,6]. The C-terminal CC motif contains six coiled-coil heptad repeats that facilitate its self-interaction [7]. Utilizing this feature, Hu *and colleagues* fused several subunits of the transcription-activator-like effector (TALE)-based tool CyDENT with γb, effectively reassembling the TALE complex in cells and significantly enhancing genome editing efficiency [8]. The BSMV γb protein exhibits diverse subcellular localization during virus infection, including the cytosol, chloroplast, plasma membrane, endoplasmic reticulum (ER), actin filaments, and plasmodesmata (PD). Considerable advances have been achieved in characterizing the novel functions of the BMSV γb proteins in the past few years (Fig 3). In this review, we will provide a detailed discussion of the current understanding of the versatile γb protein in plant-BSMV interactions.

## Multifaced roles of γb in the viral infection cycle

### γb as pathogenicity determinant

More than 30 years ago, the γb protein was demonstrated to be a pathogenicity determinant [6,9]. BSMV γb-deficient mutant substantially decreases BSMV pathogenicity in barley and *N. benthamiana* [4,10,11], and recombinant viral mutants lacking γb impair viral pathogenesis in barley and *C. amaranticolor* [6,12]. However, no noticeable growth phenotype is observed in γb transgenic *N. benthamiana* plants [4].

### γb functions in seed transmission

As one of the most well-known viruses transmitted by seeds, BSMV is hypothesized to have spread from a site near the Nile River in modern-day Egypt since approximately 750 years ago through commercial transactions [13]. In 1995, Edwards identified a 369-nt repeat in the γa gene and the proline-63 residue of γb protein from the BSMV ND18 strain as the location of the major genetic determinants of seed transmission phenotype (Fig 2A) [14]. Leveraged this characteristic, a guide RNA for CRISPR/Cas9-mediated targeted mutagenesis can be inserted into the viral genome to achieve a transgenerational gene editing in monocots [15,16].

### γb optimizes BSMV chloroplast-targeted replication

As early as the 1970s, two groups independently found that BSMV infected barley leaf cells contain numerous vesiculated chloroplast with virions surrounding these abnormally chloroplasts [17,18]. Then Lin and Langenberg found that the vesiculated proplastids of BSMV early-infected wheat cells contain viral double-stranded (ds) RNAs [19]. Subsequently, Torrance *and colleagues* also observed the deformational chloroplasts in BSMV-infected barley and *N. benthamiana* cells. Whilst they found that the γb protein can be recruited around the chloroplasts in the presence of viral RNAα and RNAγ (the minimal replication unit) [20]. These results support the notion whereby the proplastids/chloroplasts might have a role in BSMV replication and established a putative link between γb protein and the chloroplast; however, there was still no directly evidence to demonstrate whether chloroplasts are the BSMV replication sites.

**Fig 4:**
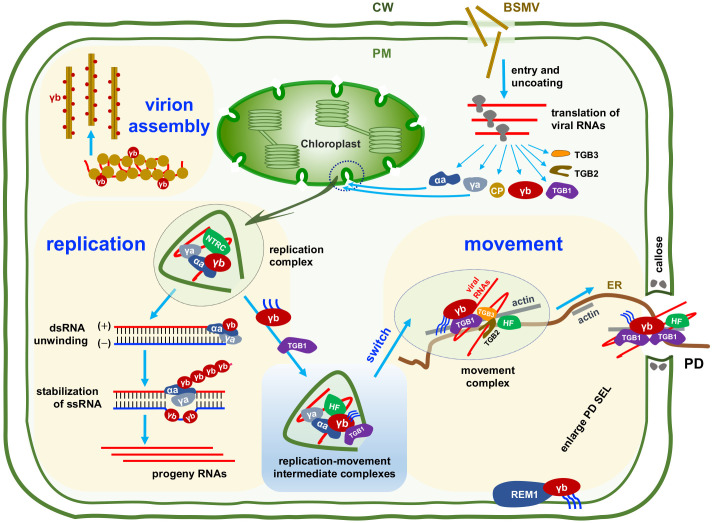
A simplified model to show the involvement of γb at different stages of BSMV infection.

Until 2017, Zhang *and colleagues* identified that the BSMV replicase subunits (αa and γa), plus- and minus-strand RNAs, as well as dsRNA replicative intermediates were all colocalized on the chloroplasts [4]. Zhang *and colleagues* also found that the γb protein was recruited to the chloroplast replication sites by direct interaction with the replicase αa, promoting its helicase activity to favor viral replication (Fig 2C) [4]. During the process of replication, γb binds to and stabilizes the single-stranded viral RNA unwound by αa and acts as a single-stranded DNA-binding (SSB)-like protein in host DNA replication process (Fig 4). Subsequently, Jin and colleagues characterized the chloroplasts outer membrane invaginated vesicles (approximately 50 nm) are the sites for BSMV replication by using three-dimensional (3D) electron tomography approach, and clarified the critical role of BSMV αa in chloroplast aberrant deformation [21].

### γb promotes BSMV movement

Petty and colleagues found that viruses with mutated γb protein exhibit a long-distance movement defect in barley [3], and this mutation also attenuates the efficiency of systemic movement in *N. benthamiana* [22]. Subsequently, Yelina *and colleagues* reported that the long-distance movement functions of potyviral HC-Pro complement the systemic movement defect of BSMV γb-deficient mutant in *N. benthamiana* [23]. In the follow-up studies, γb is consistently thought to be a long-distance movement factor.

Owing to the lack of a powerful system in long-distance movement studies, the research interests on the field of BSMV movement was gradually turned to the BSMV-encoded triple gene blocks (TGBs) movement proteins. In the early 21st century, a number of exciting research results on TGBs were discovered [24–29], and the concept of TGB modules participating in BSMV intra- and intercellular movement was basically established [30]. Subsequently, BSMV duplex fluorescence system (dfBSMV) was developed [29], which is the milestone in the study of BSMV cell-to-cell movement. In this system, an mCherry protein is co-expressed with the virus in initially inoculated cells; however, it does not move along with the GFP-labeled virus. By using this system, Li and colleagues found that the TGB1 nuclear-cytoplasmic trafficking is required for BSMV cell-to-cell movement [29].

Considering that γb interacts directly with TGB1 *in vitro* and *in vivo* (Fig 2C), the involvement of γb in TGBs-mediated cell-to-cell movement has been hypothesized. During virus infection, γb forms complexes with TGB1 at the periphery of chloroplasts and PD [10]. The dfBSMV reporter system clearly showed the requirement of γb in efficient BSMV cell-to-cell movement. Biochemical experiments revealed a novel function of γb in enhancing the ATPase activity of TGB1, thereby promoting the assembly of viral ribonucleoprotein movement complex (vRNP) [10]. The ATPase activity of TGB1 proteins encoded by *Potato virus X* (PVX) and *Beet necrotic yellow vein virus* (BNYVV) function in a similar manner to BSMV TGB1 [10], which raises an interesting question whether these viruses encode γb-like proteins promote viral movement process (Fig 4).

### γb switches viral replication to movement

It is generally acknowledged that viral replication and movement are dynamically linked, however, how plant viruses switch from active replication to movement is still unclear. Given that γb promotes BSMV replication and movement via interacting with αa and TGB1, respectively [4,10] (Fig 2C), an open question is how γb orchestrates these two completely different viral processes in one cell. Palmitoylation, also known as *S-*acylation, is a reversible lipid modification that confers membrane affinity to the modified protein and regulates its activity, enabling the protein to acquire new functions. Recently, Yue and colleagues found that *N. benthamiana S*-acyl transferase 15 (NbPAT15) and NbPAT21 interact with and palmitoylate γb protein at Cys-10, Cys-19, and Cys-60 (Fig 2A); non-palmitoylated γb localizes at chloroplasts and enhances viral replication, whereas palmitoylated γb recruits viral movement protein TGB1 to the viral replication complexes (VRCs) and facilitates the assembly of the virus movement complex [31]. In addition, palmitoylated γb further suppresses callose deposition via interactions with remorin protein to optimize BSMV intercellular movement (Fig 4). Together, this study indicates the essential role of palmitoylation in the dynamic conversion of BSMV from replication to movement [31].

### γb orchestrates BSMV virion morphogenesis

The process of virus assembly involves the coordinated action of viral structural and non-structural proteins as well as host factors and cellular organelles. However, this complicated process is still not fully clear, especially for plant viruses. By using BSMV as a model system, Yue and colleagues found that the γb protein facilitates BSMV virion morphogenesis in a Zn^2+^-dependent manner [32]. The γb protein binds to the surface of the rod-shaped BSMV particles most likely via its CCCH motif (Figs 1A and 2B), and this physical interaction enhances the RNA binding capacity of CP proteins, which in turn enhancing the assembly and stability of the viral particle (Fig 4). Given the conserved CCCH motif among *Virgaviridae* and *Benyviridae* CRPs and the detection of these proteins on their respective viral particles [32], this suggests that the convergent evolution of these proteins may have occurred during the morphogenesis of rod-shaped viruses.

### γb manipulates host defense responses

Plant have evolved multi-layered defensive pathways to perceive and defend themselves against plant virus attacks, such as autophagy [33], phytohormones transduction [34], RNA silencing [35], ubiquitin-proteasome system [36], oxidative stress and antioxidative defense [37,38], *R* gene-mediated defense [39,40], etc. In this section, we aim to summarize the current knowledge of BSMV γb protein in modulating plant defense responses.

### γb as a suppressor of RNA silencing

More than two decades ago, hordeiviral γb was identified as a viral suppressor of RNA silencing (VSR) [23]. *Cis*- or *trans*- rescue with other viral VSR proteins, such as potyviral HC-Pro and *Tomato bushy stunt virus* (TBSV) P19, partially complement BSMV accumulation [4], suggesting VSR activity is essential for BSMV pathogenesis.

**Fig 5:**
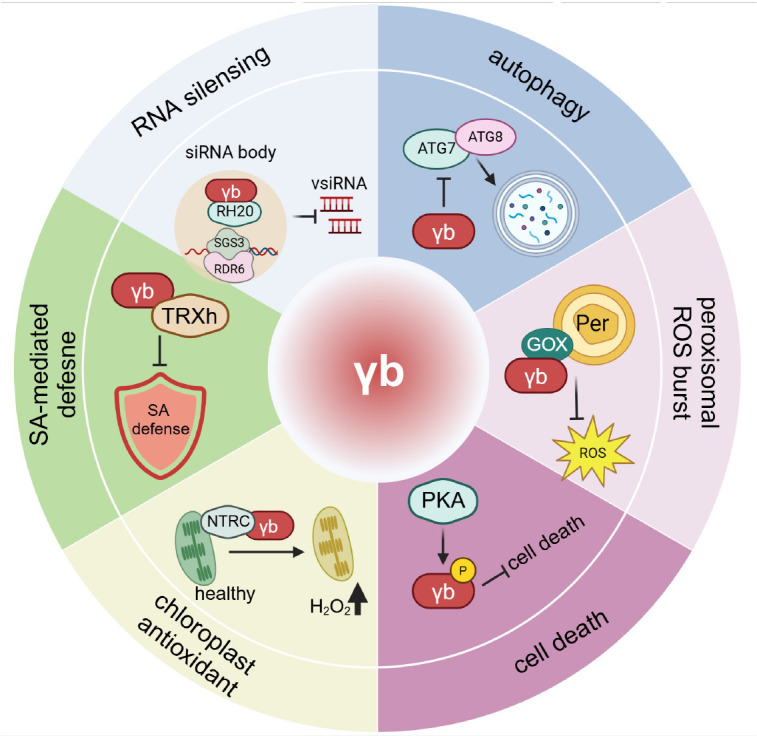
γb manipulates host defense responses. During BSMV infection, the γb protein targets to various proteins to disturb host antiviral defense, such as SA signaling pathway, RNA silencing, autophagy, ROS burst, and cell death, which, in turn, facilitating virus infection. TRXh: thioredoxin h-type; RH20: DEAD-box RNA helicase 20; SGS3: Suppressor of Gene Silencing 3; RDR6: RNA-Dependent RNA Polymerase 6; Per: Peroxisome; GOX: Glycolate oxidase; PKA: Protein kinase A; NTRC: NADPH-dependent thioredoxin reductase C. Created with BioRender.com.

γb binds ss and dsRNAs via different motifs [41]. γb suppresses the RNA silencing pathway via its N-terminal small dsRNA binding activity [42], which is regulated by phosphorylation [43]. In addition, disrupting γb homotypic interactions by inserting mutations within the C-terminal CC motif also abolish its VSR activity [7]. Intriguingly, many mutations within C1 and C2 clusters within γb also affect its VSR activity [6,41], suggesting a sophisticated modulation of γb on RNA silencing suppression (Fig 2A). Recently, Wen *and colleagues* reported that γb impairs the Asp-Glu-Ala-Asp (DEAD)-box RNA helicase 20 (RH20)-mediated antiviral RNA silencing pathway by abolishing the RH20-Suppressor of Gene Silencing 3 (SGS3)-RNA-Dependent RNA polymerase 6 (RDR6) complex (Fig 5) [44]. However, the overall understanding of the function of γb in RNA silencing pathway remains incomplete.

In the past 8 years, beyond RNA silencing suppressor, a series of novel functions of the γb proteins have been deciphered in modulating virus infection cycles and counteracting host antiviral defenses for optimize BSMV infections.

### γb subverts autophagy

Autophagy is emerging as an essential defense strategy for plants [33,45,46]. Silencing of the key *autophagy-related gene 5* (*ATG5*) and *ATG7* significantly increases BSMV accumulation in *N. benthamiana*, suggesting the autophagy pathway may also play an antiviral role during BSMV infection [47]. To counteract autophagy-mediated defenses, the γb protein competitively inhibits the interaction of ATG7 with ATG8 by directly targeting to ATG7, thereby subverting the autophagy pathway (Fig 5). A point mutation within γb, Y29A, abolishing its affinity to ATG7 led to reduced viral symptoms and viral accumulation in upper leaves of *N. benthamiana* (Fig 2A) [47]. In addition, Yang *and colleagues* recently reported that the BSMV-encoded replicase γa suppresses autophagy via blocking vacuolar acidification [48]. The γa protein disrupts the interaction between V-ATPase catalytic (VHA) subunit B2 and VHA subunit E, resulting in the release of VHA-B2 from the tonoplast and reducing V-ATPase activity, which inhibit vacuolar acidification and cargo degradation in the vacuole [48]. These results show that a BSMV may employ distinct strategies to cooperatively inhibit the autophagy-mediated defense.

### γb disturbs peroxisomal ROS burst

Reactive oxygen species (ROS) are one of the earliest cellular response signals against stresses. BSMV infection triggers ROS burst in *N. benthamiana*, however, BSMV γb protein enable to decrease the level of ROS by >90%. Molecular analyses indicate that γb interacts with and inhibits the glycolate oxidase (GOX), a peroxisomal enzyme which generates glyoxylate and H_2_O_2_ during photorespiration, to reduce peroxisomal ROS production and facilitate BSMV infections (Fig 5) [49].

### γb suppresses cell death

In addition to palmitoylation modification, BSMV γb is subjected to phosphorylation at its Ser-96 by a protein kinase A (PKA)-like kinase (Fig 2A). Phospho-dead mutants of γb triggers cell death and decreases viral accumulation and systemic infection in *N. benthamiana*, barley, and wheat, whereas the mimic-phosphorylated γb mutant exhibits similar pathogenesis compared to wild-type virus [43]. Intriguingly, phosphorylation of γb enhances its VSR activity, as evidenced by a strong affinity to 21-bp dsRNA, albeit the VSR activity is functionally distinct from cell death suppression (Fig 5) [43].

### γb impairs chloroplast antioxidant defense

Chloroplast antioxidant defense is an important strategy for plant in response to diverse abiotic and biotic stresses [50]. In order to avoid oxidative damages in cells, plant have evolved a series of antioxidant mechanisms to balance the ROS production and scavenging [51]. In addition to suppress the peroxisomal ROS production, Wang *and colleagues* found that the BSMV γb protein disrupts chloroplasts antioxidant defenses. Molecular assays showed that γb interacts directly with NADPH-dependent thioredoxin reductase C (NTRC), a core component involved in chloroplast antioxidant defense, and competitively interferes with NTRC targeting to 2-Cys Prx for ROS scavenging in chloroplasts, which in turn creates an oxidized microenvironments at BSMV replication site conducive to BSMV replication (Fig 5) [38].

### γb interferes with salicylic acid-mediated defense

Salicylic acid (SA) acts as a defense hormone for perception and protection against distinct pathogens [52]. BSMV infection also triggers the SA defense signaling pathway in *N. benthamiana* [53]. To counteract this antiviral defense, the BSMV γb protein downregulates the downstream SA defense genes expression by interacting with *N. benthamiana* thioredoxin h-type 1 (NbTRXh1), an orthologue of AtTRXh3 and AtTRXh5 catalyzing the SA signaling transduction in *Arabidopsis* [54]. Overexpression of NbTRXh1, but not its reductase-defective mutant, inhibits BSMV infections, whereas silencing *NbTRXh1* increases BSMV pathogenesis. Genetic assays showed that γb transgenic *N. benthamiana* plants display lower *pathogenesis-related gene 1* (*PR1*) and *PR2* gene expression compared with non-transgenic plants. *In vitro* biochemistry assays revealed that γb interferes directly with the reductase activity of NbTRXh1, thereby blocking SA signaling transduction to promote BSMV infection (Fig 5) [53].

## The Achilles’ heel of γb

The widespread viral multifunctional proteins prompt the questions of how plants adequately respond to viral infection. Host plants have also evolved sophisticated counter-counter-defense strategies against constant pathogens’ attack, which, in turn, maintain a dynamic selection pressure underpinning the continuous co-evolution between plants and viruses.

Although the versatile roles of γb in counteracting plant defense, Zhang *and colleagues* found that the autophosphorylated cytosolic serine/threonine/tyrosine protein kinase 46 of *N. benthamiana* (NbSTY46) can inhibit BSMV replication and alleviate BSMV virulence via phosphorylation of the γb proteins [55]. Furthermore, Li and colleagues reported that the *S*-adenosylmethionine decarboxylase 3 (SAMDC3) protein reduces the BSMV infectivity by targeting γb and promoting its degradation through the proteasome via increased ubiquitination [56]. These results suggest that regulating the post-translational modifications of viral proteins could serve as an antiviral strategy in the co-evolutionary arms race between host plants and invading viruses.

## Concluding remarks and future perspectives

One of the substantial differences between viruses and other pathogens is that most viruses do not have the luxury of genome duplication. To establish successful infections, viruses also require the same array of effectors functions as other pathogens group; therefore, each viral protein must execute multiple roles in the infection cycle as well as counteract plant defenses. Multifunctional proteins usually show multiple subcellular localizations; the BSMV γb protein mainly distributes in the cytoplasm when transiently expressed in *N. benthamiana* epidermal cells; however, in the context of the viral infection, it can be targeted to the chloroplasts, peroxisomes, cytosol, ER, actin, plasma membrane, and plasmodesmata at different stages of infection [30]. By localizing to different subcellular compartments, one viral protein can gain a novel interactome landscape. To date, the γb protein has been shown to physically interact with itself and the essential proteins required for BSMV full infection (replicase, movement protein, and CP) [30,32] as well as large amounts of identified or potential host proteins to carry out versatile functions during BSMV infections [44]. These intricate interaction networks have contributed to our overall understanding of the virus infections. On the other hand, the crystal structure of the γb protein has not been resolved yet due to the N-terminal disordered region, the solving of this challenge will provide a perfect explanation for the functional diversity of the γb protein.

## Abbreviations

ALBV: *Anthoxanthum latent blanching virus*
BSMV: *Barley stripe mosaic virus*
CRPs: cysteine-rich proteins
LigMV: *Ligustrum mosaic virus*
LRSV: *Lychnis ringspot virus*
PSLV: *Poa semilatent virus*
ROS: reactive oxygen species
TALE: transcription-activator-like effector
TGB: triple gene block

## References

[1] Adams MJ, Adkins S, Bragard C, Gilmer D, Li D, MacFarlane SA, et al. ICTV virus taxonomy profile: *Virgaviridae*. J Gen Virol. 2017;98(8):1999–2000. doi: 10.1099/jgv.0.000884 . 28786782 PMC5656781

[2] Reynard J-S, Turco S, Brodard J, Kellenberger I, Maclot F, Schumpp O, et al. Identification and molecular characterization of a novel *Hordeivirus* associated with yellow mosaic disease of privet (*Ligustrum vulgare*) in Europe. Front Microbiol. 2021;12:723350. doi: 10.3389/fmicb.2021.723350 . 34646247 PMC8503643

[3] Petty IT, French R, Jones RW, Jackson AO. Identification of *Barley stripe mosaic virus* genes involved in viral RNA replication and systemic movement. EMBO J. 1990;9(11):3453–7. doi: 10.1002/j.1460-2075.1990.tb07553.x . 2209552 PMC552093

[4] Zhang K, Zhang Y, Yang M, Liu S, Li Z, Wang X, et al. The *Barley stripe mosaic virus* γb protein promotes chloroplast-targeted replication by enhancing unwinding of RNA duplexes. PLoS Pathog. 2017;13(4):e1006319. doi: 10.1371/journal.ppat.1006319 . 28388677 PMC5397070

[5] Bragg JN, Lawrence DM, Jackson AO. The N-terminal 85 amino acids of the *Barley stripe mosaic virus* γb pathogenesis protein contain three zinc-binding motifs. J Virol. 2004;78(14):7379–91. doi: 10.1128/JVI.78.14.7379-7391.2004 . 15220411 PMC434125

[6] Donald RG, Jackson AO. The *Barley stripe mosaic virus* γb gene encodes a multifunctional cysteine-rich protein that affects pathogenesis. Plant Cell. 1994;6(11):1593–606. doi: 10.1105/tpc.6.11.1593 . 7827493 PMC160546

[7] Bragg JN, Jackson AO. The C-terminal region of the *Barley stripe mosaic virus* γb protein participates in homologous interactions and is required for suppression of RNA silencing. Mol Plant Pathol. 2004;5(5):465–81. doi: 10.1111/j.1364-3703.2004.00246.x . 20565621

[8] Hu J, Sun Y, Li B, Liu Z, Wang Z, Gao Q, et al. Strand-preferred base editing of organellar and nuclear genomes using CyDENT. Nat Biotechnol. 2024;42(6):936–45. doi: 10.1038/s41587-023-01910-9 . 37640945

[9] Jackson AO, Petty ITD, Jones RW, Edwards MC, French R. Molecular genetic analysis of *Barley stripe mosaic virus* pathogenicity determinants. Can J Plant Pathol. 1991;13(2):163–77. doi: 10.1080/07060669109500952

[10] Jiang Z, Zhang K, Li Z, Li Z, Yang M, Jin X, et al. The *Barley stripe mosaic virus* γb protein promotes viral cell-to-cell movement by enhancing ATPase-mediated assembly of ribonucleoprotein movement complexes. PLoS Pathog. 2020;16(7):e1008709. doi: 10.1371/journal.ppat.1008709 . 32730331 PMC7419011

[11] Jackson AO, Lim H-S, Bragg J, Ganesan U, Lee MY. *Hordeivirus* replication, movement, and pathogenesis. Annu Rev Phytopathol. 2009;47:385–422. doi: 10.1146/annurev-phyto-080508-081733 . 19400645

[12] Petty IT, Donald RG, Jackson AO. Multiple genetic determinants of *Barley stripe mosaic virus* influence lesion phenotype on *Chenopodium amaranticolor*. Virology. 1994;198(1):218–26. doi: 10.1006/viro.1994.1024 . 8259657

[13] Smith O, Clapham A, Rose P, Liu Y, Wang J, Allaby RG. A complete ancient RNA genome: identification, reconstruction and evolutionary history of archaeological *Barley stripe mosaic virus*. Sci Rep. 2014;4:4003. doi: 10.1038/srep04003 . 24499968 PMC3915304

[14] Edwards MC. Mapping of the seed transmission determinants of *Barley stripe mosaic virus*. Mol Plant Microbe Interact. 1995;8(6):906–15. doi: 10.1094/mpmi-8-0906 . 8664501

[15] Hu J, Li S, Li Z, Li H, Song W, Zhao H, et al. A *Barley stripe mosaic virus*-based guide RNA delivery system for targeted mutagenesis in wheat and maize. Mol Plant Pathol. 2019;20(10):1463–74. doi: 10.1111/mpp.12849 . 31273916 PMC6792137

[16] Li T, Hu J, Sun Y, Li B, Zhang D, Li W, et al. Highly efficient heritable genome editing in wheat using an RNA virus and bypassing tissue culture. Mol Plant. 2021;14(11):1787–98. doi: 10.1016/j.molp.2021.07.010 . 34274523

[17] Carroll TW. Relation of *Barley stripe mosaic virus* to plastids. Virology. 1970;42(4):1015–22. doi: 10.1016/0042-6822(70)90350-8 . 4099076

[18] McMullen CR. Aberrant plastids in barley leaf tissue infected with *Barley stripe mosaic virus*. Phytopathology. 1978;68(3):317. doi: 10.1094/phyto-68-317

[19] Lin NS, Langenberg WG. Peripheral vesicles in proplastids of *Barley stripe mosaic virus*-infected wheat cells contain double-stranded RNA. Virology. 1985;142(2):291–8. doi: 10.1016/0042-6822(85)90337-x . 18639846

[20] Torrance L, Cowan GH, Gillespie T, Ziegler A, Lacomme C. *Barley stripe mosaic virus*-encoded proteins triple-gene block 2 and γb localize to chloroplasts in virus-infected monocot and dicot plants, revealing hitherto-unknown roles in virus replication. J Gen Virol. 2006;87(Pt 8):2403–11. doi: 10.1099/vir.0.81975-0 . 16847137

[21] Jin X, Jiang Z, Zhang K, Wang P, Cao X, Yue N, et al. Three-dimensional analysis of chloroplast structures associated with virus infection. Plant Physiol. 2018;176(1):282–94. doi: 10.1104/pp.17.00871 . 28821590 PMC5761806

[22] Petty IT, Edwards MC, Jackson AO. Systemic movement of an RNA plant virus determined by a point substitution in a 5′ leader sequence. Proc Natl Acad Sci U S A. 1990;87(22):8894–7. doi: 10.1073/pnas.87.22.8894 . 2247462 PMC55066

[23] Yelina NE, Savenkov EI, Solovyev AG, Morozov SY, Valkonen JPT. Long-distance movement, virulence, and RNA silencing suppression controlled by a single protein in hordei- and potyviruses: complementary functions between virus families. J Virol. 2002;76(24):12981–91. doi: 10.1128/jvi.76.24.12981-12991.2002 . 12438624 PMC136670

[24] Lawrence DM, Jackson AO. Interactions of the TGB1 protein during cell-to-cell movement of *Barley stripe mosaic virus*. J Virol. 2001;75(18):8712–23. doi: 10.1128/jvi.75.18.8712-8723.2001 . 11507216 PMC115116

[25] Lim H-S, Bragg JN, Ganesan U, Lawrence DM, Yu J, Isogai M, et al. Triple gene block protein interactions involved in movement of *Barley stripe mosaic virus*. J Virol. 2008;82(10):4991–5006. doi: 10.1128/JVI.02586-07 . 18353960 PMC2346763

[26] Lim H-S, Bragg JN, Ganesan U, Ruzin S, Schichnes D, Lee MY, et al. Subcellular localization of the *Barley stripe mosaic virus* triple gene block proteins. J Virol. 2009;83(18):9432–48. doi: 10.1128/JVI.00739-09 . 19570874 PMC2738231

[27] Lim H-S, Lee MY, Moon JS, Moon J-K, Yu Y-M, Cho IS, et al. Actin cytoskeleton and Golgi involvement in *Barley stripe mosaic virus* movement and cell wall localization of triple gene block proteins. Plant Pathol J. 2013;29(1):17–30. doi: 10.5423/PPJ.OA.09.2012.0144 . 25288925 PMC4174794

[28] Hu Y, Li Z, Yuan C, Jin X, Yan L, Zhao X, et al. Phosphorylation of TGB1 by protein kinase CK2 promotes *Barley stripe mosaic virus* movement in monocots and dicots. J Exp Bot. 2015;66(15):4733–47. doi: 10.1093/jxb/erv237 . 25998907 PMC4507770

[29] Li Z, Zhang Y, Jiang Z, Jin X, Zhang K, Wang X, et al. Hijacking of the nucleolar protein fibrillarin by TGB1 is required for cell-to-cell movement of *Barley stripe mosaic virus*. Mol Plant Pathol. 2018;19(5):1222–37. doi: 10.1111/mpp.12612 . 28872759 PMC6638131

[30] Jiang Z, Yang M, Zhang Y, Jackson AO, Li D. Hordeiviruses (*Virgaviridae*). In: Bamford DH, Zuckerman M, eds. Encyclopedia of Virology. Fourth ed. Oxford: Academic Press; 2021. p. 420–9. doi: 10.1016/b978-0-12-809633-8.21250-8

[31] Yue N, Jiang Z, Zhang X, Li Z, Wang X, Wen Z, et al. Palmitoylation of γb protein directs a dynamic switch between *Barley stripe mosaic virus* replication and movement. EMBO J. 2022;41(13):e110060. doi: 10.15252/embj.2021110060 . 35642376 PMC9251889

[32] Yue N, Jiang Z, Pi Q, Yang M, Gao Z, Wang X, et al. Zn^2+^-dependent association of cysteine-rich protein with virion orchestrates morphogenesis of rod-shaped viruses. PLoS Pathog. 2024;20(6):e1012311. doi: 10.1371/journal.ppat.1012311 . 38885273 PMC11213338

[33] Yang M, Ismayil A, Liu Y. Autophagy in plant–virus interactions. Annu Rev Virol. 2020;7(1):403–19. doi: 10.1146/annurev-virology-010220-054709 . 32530794

[34] Zhao S, Li Y. Current understanding of the interplays between host hormones and plant viral infections. PLoS Pathog. 2021;17(2):e1009242. doi: 10.1371/journal.ppat.1009242 . 33630970 PMC7906326

[35] Lopez-Gomollon S, Baulcombe DC. Roles of RNA silencing in viral and non-viral plant immunity and in the crosstalk between disease resistance systems. Nat Rev Mol Cell Biol. 2022;23(10):645–62. doi: 10.1038/s41580-022-00496-5 . 35710830

[36] Wu J, Zhang Y, Li F, Zhang X, Ye J, Wei T, et al. Plant virology in the 21st century in China: recent advances and future directions. J Integr Plant Biol. 2024;66(3):579–622. doi: 10.1111/jipb.13580 . 37924266

[37] Hernández JA, Gullner G, Clemente-Moreno MJ, Künstler A, Juhász C, Díaz-Vivancos P, et al. Oxidative stress and antioxidative responses in plant–virus interactions. Physiol Mol Plant Pathol. 2016;94:134–48. doi: 10.1016/j.pmpp.2015.09.001

[38] Wang X, Jiang Z, Yue N, Jin X, Zhang X, Li Z, et al. *Barley stripe mosaic virus* γb protein disrupts chloroplast antioxidant defenses to optimize viral replication. EMBO J. 2021;40(16):e107660. doi: 10.15252/embj.2021107660 . 34254679 PMC8365260

[39] Zhu M, Feng M, Tao X. NLR-mediated antiviral immunity in plants. J Integr Plant Biol. 2025;67(3):786–800. doi: 10.1111/jipb.13821 . 39777907

[40] Sett S, Prasad A, Prasad M. Resistance genes on the verge of plant-virus interaction. Trends Plant Sci. 2022;27(12):1242–52. doi: 10.1016/j.tplants.2022.07.003 . 35902346

[41] Donald RG, Jackson AO. RNA-binding activities of *Barley stripe mosaic virus* γb fusion proteins. J Gen Virol. 1996;77 ( Pt 5):879–88. doi: 10.1099/0022-1317-77-5-879 . 8609484

[42] Mérai Z, Kerényi Z, Kertész S, Magna M, Lakatos L, Silhavy D. Double-stranded RNA binding may be a general plant RNA viral strategy to suppress RNA silencing. J Virol. 2006;80(12):5747–56. doi: 10.1128/JVI.01963-05 . 16731914 PMC1472586

[43] Zhang X, Dong K, Xu K, Zhang K, Jin X, Yang M, et al. *Barley stripe mosaic virus* infection requires PKA-mediated phosphorylation of γb for suppression of both RNA silencing and the host cell death response. New Phytol. 2018;218(4):1570–85. doi: 10.1111/nph.15065 . 29453938

[44] Wen Z, Hu R, Pi Q, Zhang D, Duan J, Li Z, et al. DEAD-box RNA helicase RH20 positively regulates RNAi-based antiviral immunity in plants by associating with SGS3/RDR6 bodies. Plant Biotechnol J. 2024;22(12):3295–311. doi: 10.1111/pbi.14448 . 39166471 PMC11606427

[45] Sertsuvalkul N, DeMell A, Dinesh-Kumar SP. The complex roles of autophagy in plant immunity. FEBS Lett. 2022;596(17):2163–71. doi: 10.1002/1873-3468.14356 . 35460270 PMC9474723

[46] Yang M, Liu Y. Autophagy in plant viral infection. FEBS Lett. 2022;596(17):2152–62. doi: 10.1002/1873-3468.14349 . 35404481

[47] Yang M, Zhang Y, Xie X, Yue N, Li J, Wang X-B, et al. *Barley stripe mosaic virus* γb protein subverts autophagy to promote viral infection by disrupting the ATG7-ATG8 interaction. Plant Cell. 2018;30(7):1582–95. doi: 10.1105/tpc.18.00122 . 29848767 PMC6096602

[48] Yang M, Ismayil A, Jiang Z, Wang Y, Zheng X, Yan L, et al. A viral protein disrupts vacuolar acidification to facilitate virus infection in plants. EMBO J. 2022;41(2):e108713. doi: 10.15252/embj.2021108713 . 34888888 PMC8762549

[49] Yang M, Li Z, Zhang K, Zhang X, Zhang Y, Wang X, et al. *Barley stripe mosaic virus* γb interacts with glycolate oxidase and inhibits peroxisomal ROS production to facilitate virus infection. Mol Plant. 2018;11(2):338–41. doi: 10.1016/j.molp.2017.10.007 . 29066357

[50] Moustakas M, Sperdouli I, Adamakis I-DS. Editorial: Reactive oxygen species in chloroplasts and chloroplast antioxidants under abiotic stress. Front Plant Sci. 2023;14:1208247. doi: 10.3389/fpls.2023.1208247 . 37304709 PMC10254792

[51] Gill SS, Tuteja N. Reactive oxygen species and antioxidant machinery in abiotic stress tolerance in crop plants. Plant Physiol Biochem. 2010;48(12):909–30. doi: 10.1016/j.plaphy.2010.08.016 . 20870416

[52] Spoel SH, Dong X. Salicylic acid in plant immunity and beyond. Plant Cell. 2024;36(5):1451–64. doi: 10.1093/plcell/koad329 . 38163634 PMC11062473

[53] Jiang Z, Jin X, Yang M, Pi Q, Cao Q, Li Z, et al. *Barley stripe mosaic virus* γb protein targets thioredoxin h-type 1 to dampen salicylic acid-mediated defenses. Plant Physiol. 2022;189(3):1715–27. doi: 10.1093/plphys/kiac137 . 35325212 PMC9237698

[54] Tada Y, Spoel SH, Pajerowska-Mukhtar K, Mou Z, Song J, Wang C, et al. Plant immunity requires conformational changes [corrected] of NPR1 via S-nitrosylation and thioredoxins. Science. 2008;321(5891):952–6. doi: 10.1126/science.1156970 . 18635760 PMC3833675

[55] Zhang X, Wang X, Xu K, Jiang Z, Dong K, Xie X, et al. The serine/threonine/tyrosine kinase STY46 defends against *Hordeivirus* infection by phosphorylating γb protein. Plant Physiol. 2021;186(1):715–30. doi: 10.1093/plphys/kiab056 . 33576790 PMC8154058

[56] Li Z, Yang X, Li W, Wen Z, Duan J, Jiang Z, et al. SAMDC3 enhances resistance to *Barley stripe mosaic virus* by promoting the ubiquitination and proteasomal degradation of viral γb protein. New Phytol. 2022;234(2):618–33. doi: 10.1111/nph.17993 . 35075654

